# Genetic and Haplotype Diversity of *Schizopygopsis pylzovi* in the Yellow River on the Northeastern Qinghai–Tibet Plateau

**DOI:** 10.3390/ani16131946

**Published:** 2026-06-23

**Authors:** Qunhui Xiao, Xinyu Qu, Hongyan Liu, Zixia Zhao, Ran Zhao, Jin Zhang, Yanliang Jiang

**Affiliations:** 1 College of Fisheries and Life Science, Shanghai Ocean University, Shanghai 201306, China; 2 Key Laboratory of Aquatic Genomics, Ministry of Agriculture and Rural Affairs, CAFS Key Laboratory of Aquatic Genomics, Chinese Academy of Fishery Sciences, Beijing 100141, China

**Keywords:** *Schizopygopsis pylzovi*, genetic diversity, population structure, mitochondrial DNA, molecular markers

## Abstract

The uplift of the Qinghai–Tibet Plateau has shaped a unique extreme environment, fostering distinct endemic aquatic organisms. *Schizopygopsis pylzovi*, a vulnerable endemic fish in the upper Yellow River, is a key model for studying the biogeographic patterns of plateau fish. To assess its genetic characteristics and evolutionary dynamics, we comprehensively evaluated 11 geographic populations of *S. pylzovi* using two complementary mitochondrial markers, the conserved COI gene and the fast-evolving D-loop region. A total of 142 COI and 143 D-loop sequences were analyzed, and sequences alignment, haplotype network construction, AMOVA, and neutrality tests were performed. AMOVA revealed that genetic variation was mainly distributed within populations, indicating weak population differentiation. Neutrality tests and mismatch distribution analysis suggested historical and recent population expansion events. Our findings highlight the value of joint analysis using COI and D-loop markers in revealing the genetic structure of *S. pylzovi*, provide new insights into the impact of plateau uplift on fish evolution, and establish a scientific basis for the conservation of this vulnerable species.

## Simple Summary

The upper Yellow River on the Qinghai–Tibet Plateau is home to a unique fish species, the vulnerable *Schizopygopsis pylzovi*. Understanding its genetic status is essential for preventing the extinction of this species. In this study, researchers collected fish samples from 11 different locations along the river and analyzed two maternally inherited mitochondrial DNA fragments: the rapidly evolving and highly polymorphic D-loop region, and the relatively conserved COI gene. The results showed that most genetic variation resides within local populations rather than among different populations, indicating that fish from different geographical areas may interbreed and exchange genes. However, certain natural hydrological barriers may lead to subtle genetic differentiation among some populations. In addition, some populations suggested signs of past population expansion or bottleneck events. These findings provide a scientific basis for conservation managers to make informed decisions to protect this vulnerable fish species and its riverine habitat.

## 1. Introduction

The uplift of the Qinghai–Tibet Plateau is of great significance for East Asian water systems and biological evolution. Over millions of years, this uplift has altered and complicated the hydrological patterns, resulting in a unique environment characterized by low temperatures, low oxygen levels, high salinity, and intense ultraviolet radiation [1], which in turn has fostered a distinct and diverse freshwater fish fauna. Within this evolutionary context, the adaptive evolution of species in the subfamily Schizothoracinae serves as a classic example for elucidating the biogeographic patterns of the plateau [2]. As a representative species of this subfamily, Pylzov’s schizopygopsis (*Schizopygopsis pylzovi* Kessler, 1876) possesses distinctive scientific merit and ecological significance [3]. It has been listed as a vulnerable species in the China Species Red List, and is an endemic species in the Yellow River, which is mainly distributed in the upstream of the Yellow River above an altitude of 3000 m [3]. This rare, endemic species plays a dual role in the upper Yellow River ecosystem: it is a key component of the plateau’s cold-water food web and a sensitive indicator species of changes in water quality. There is a study demonstrated that this species exhibits high genetic diversity and fulfills irreplaceable biological functions in maintaining regional aquatic biodiversity [4]. However, they concatenated the control region and cytochrome b gene sequences into a single combined dataset without separately analyzing the signals of individual markers, focused primarily on different tributary populations of the Yellow River, and did not report haplotype diversity. Consequently, a comprehensive assessment of the genetic characteristics of *S. pylzovi* using independent, complementary mitochondrial markers across the mainstream of the upper Yellow River is still lacking. An in-depth analysis of the population genetic structure of *S. pylzovi* can provide valuable insights: it not only reveals the mechanisms by which the uplift of the plateau since the Late Cenozoic has shaped the geographic patterns of fish lineages but also offers a new perspective on the adaptive evolution of significant indigenous fish species in heterogeneous habitats. This understanding has significant theoretical implications for interpreting the formation mechanisms of aquatic biodiversity on the northeastern edge of the Qinghai-Tibetan Plateau.

Molecular markers are essential and indispensable tools for the population genetic studies and phylogenetic analysis of biological taxa. Genetic tracer technology utilizing molecular markers enables investigations into evolutionary history, population structure, germplasm evaluation, and inter-population relationships, and is crucial for biological research and genetic resource investigations [5]. Mitochondrial genomic markers, due to their molecular evolutionary characteristics, are particularly valuable for assessing species diversity and reconstructing phylogenies [6]. A key focus is often on the displacement loop (D-loop) within the control region, containing numerous single nucleotide polymorphisms (SNPs) and structural variations, making it widely utilized in studies of population genetic differentiation and phylogeography [7,8]. Conversely, the cytochrome oxidase subunit I (COI) gene, a typical coding region of the mitochondrial genome, offers a different profile. Its structure is compact and its sequence is more conserved, yet it retains sufficient variation to serve as a standard barcode for species identification and broader biodiversity assessments [9].

Currently, the mitochondrial COI gene and D-loop region are widely used as complementary molecular markers for investigating genetic diversity in fish. The combined application of the COI gene and D-loop region markers can elucidate deep-time evolutionary history and reveal recent population dynamics, providing essential genetic evidence for species conservation and management. For instance, Lin et al. evaluated the genetic diversity of black bass using both mitochondrial D-loop region and COI gene sequences [10]. Mehrnoush et al. utilized both the mitochondrial COI gene and D-loop region to examine the genetic diversity of red-breasted tilapia (*Coptudon rendalli*), providing reliable evidence for conservation and management strategies [11]. Similarly, Fang et al. utilized both markers to assess the genetic diversity, population structure, and historical population dynamics of naked carp from seven geographical regions of Qinghai Lake, identifying climate change and human activities as major threats to the genetic diversity of this species [12]. In order to determine the genetic characteristics of *S. pylzovi*, in this study, a comprehensive assessment was conducted to investigate the genetic variation and differentiation, haplotype diversity, phylogenetic relationships, population structure, and historical population dynamics by integrating the dual molecular markers of the COI gene and D-loop region.

## 2. Materials and Methods

### 2.1. Ethics Statement and Sample Collection

This study was approved by the Laboratory Animal Ethics Committee of the Chinese Academy of Fishery Sciences, with the approval No. of CAFS20250426. The collection of the sampled fish in this study was permitted by the Bureau of Fisheries and Fishery Administration, Ministry of Agriculture and Rural Affairs of the People’s Republic of China, and the sample collection strictly complied with the relevant regulations of wildlife management.

**Figure 1:**
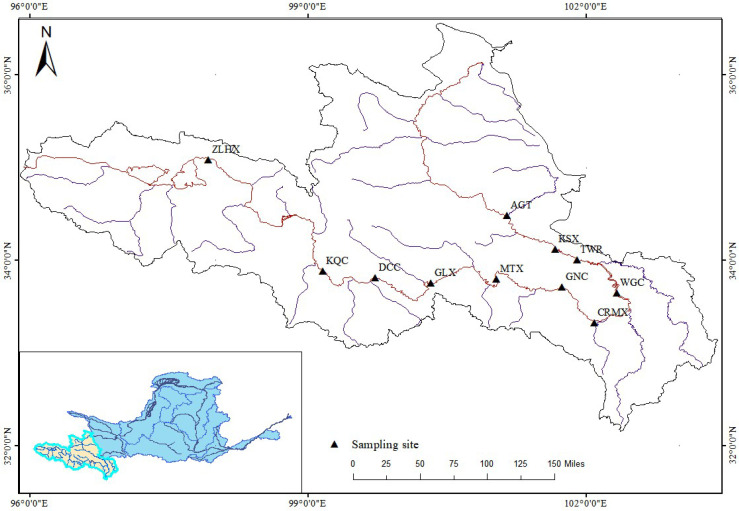
Sampling locations of *Schizopygopsis pylzovi* populations. The red line represents the main stream of the Yellow River and the purple lines represent tributaries. The black solid triangles represent sampling sites. The box in the lower left corner represents the entire Yellow River Basin.

11 geographic populations of *S. pylzovi* across the upper Yellow River Basin were collected (Figure 1), including Agetang (AGT), Cairimaxiang (CRMX), Dangchengcun (DCC), Ganglongxiang (GLX), Gongnaicun (GNC), Keshengxiang (KSX), Kequcun (KQC), Mentangxiang (MTX), Taiwuruo (TWR), Wengecun (WGC), and Zhalinghuxiang (ZLHX). These 11 sites were selected to systematically cover the known distribution range of the *S. pylzovi* in the upper Yellow River. The selection took into account habitat types, the appropriateness of spatial distances, and accessibility, while also referencing historical data from the Yellow River survey. Detailed information of the 11 sampling sites are shown in Table S1. Fin tissues from *S. pylzovi* were collected at each sampling site and immediately preserved in 95% ethanol until DNA isolation.

### 2.2. DNA Extraction, Amplification and Sequencing

Genomic DNA was extracted from fin tissue by the phenol-chloroform method [13], and high-purity DNA was obtained by proteinase K digestion, phenol/chloroform/isoamyl alcohol (25:24:1) gradient centrifugation purification, and anhydrous ethanol precipitation. After integrity detection by 1% agarose gel electrophoresis, the concentration of the DNA was determined and diluted to a working concentration of 100 ng/μL by using a NanoDrop 2000 spectrophotometer (Thermo Fisher Scientific, MA, USA). Specific primers were designed by using Primer3Plus (https://www.primer3plus.com/, accessed on 5 June 2025). The primers COI (F: 5‘-TCAACCAACCACAAAGACATTGGCAC-3′; R: 5′-TAGACTTCTGGGTGGCCAAAGAATCA-3′) and DL (F: 5′-AACCACAAAGCAAGTACTAAATTCT-3′; R: 5′-AAATTTTAGTAGGGGTTGACACG-3′) were used to amplify the mitochondrial COI region and the D-loop gene, respectively. The PCR reaction system was 50 μL, including 25 μL 2× Es Taq MasterMix (Kangwei Century, Jiangsu, China), 2 μL upstream and downstream primers, 2 μL template DNA, and 21 μL ddH_2_O. The program was set to 95 °C of pre-denaturation for 5 min, followed by 35 cycles of denaturation at 95 °C for 30 s, annealing at 57 °C (D-loop)/62 °C (COI) for 30 s, extension at 72 °C for 1 min, and final extension at 72 °C for 5 min. The annealing temperatures were initially estimated based on the calculated melting temperatures of the primers, and then experimentally optimized using gradient PCR. After electrophoresis on a 1.5% agarose gel, the amplified products were visualized under UV light. The PCR products were purified and sent to Taihe Biotechnology Co., Ltd. (Beijing, China) for sequencing.

### 2.3. Data Analysis

All sequences were verified by homology comparison with the NCBI database (https://blast.ncbi.nlm.nih.gov, accessed on 10 July 2025) based on the BLASTN searches with an E-value cutoff of 1 × 10^−5^. Sequences were aligned using MEGA 11.0 [14] software to ensure the accuracy of sequences. Haplotype phylogenetic trees were constructed using the neighbor-joining method in MEGA 11. The optimal nucleotide substitution model for each marker was determined separately using the Model Selection function with the BIC criterion; the Kimura 2-parameter (K2P) + Gamma (G) model was selected. Node support was evaluated by bootstrap analysis with 1000 replicates. The number of haplotypes (N) and the distribution of polymorphic sites (S) were counted based on the DnaSP 6 platform [15], and genetic diversity parameters including haplotype diversity (h) and nucleotide diversity (π) were also calculated. To explore the historical dynamics of the population, neutrality tests (Tajima’s D and Fu’s Fs-test) and expansion analysis were performed by Arlequin 3.5 software [16], and 10,000 random permutations were used to verify the degree of deviation of population expansion events from the neutral evolutionary model. Sequence data exported from DnaSP 6 were used in the PopART [17] program to construct the TCS haplotype network of the D-loop and COI genes to visually present the geographic distribution of haplotypes. Population genetic structure analysis was accomplished by analysis of molecular variance (AMOVA) using Arlequin software to quantitatively assess the level of genetic differentiation between populations based on paired *F*_ST_ values, and significance testing was performed by 10,000 Monte Carlo simulations [18].

## 3. Results

### 3.1. Sequence Variation and Genetic Diversity

A total of 150 specimens of *S. pylzovi* were collected from 11 geographic populations. After PCR amplification, sequencing, removal of sequences with low quality, 142 COI genes with length of 703 bp and 143 D-loop region sequences with length of 837 bp were obtained. The overall base composition of COI gene was A (24.9%), T (30.9%), G (18.2%) and C (26%), showing obvious anti-G bias. AT content (55.8%) was higher than GC content (44.2%), showing AT bias, which was consistent with other fishes [19]. The overall base composition of D-loop gene was A (30.1%), T (30.8%), G (16%) and C (23%), showing obvious anti-G bias. AT content (60.9%) was higher than GC content (39%), showing AT bias, which was consistent with other fishes [19]. The COI gene fragments yielded 14 distinct haplotypes. As shown in Table S2, Hap_2 was the dominant haplotype, which was identified in all 11 populations, with the highest frequency in TWR (20.4%) and GNC (20.4%), followed by AGT (12.9%). Hap_3 was the second most common haplotype, which was identified in nine out of 11 populations, with the highest frequency in GNC (21.7%) and KQC (21.7%), followed by AGT (13.0%). For the remaining haplotypes, Hap_7 and Hap_9 were distributed in 5 populations; Hap_6 was distributed in two populations; Hap_1, Hap_4, Hap_5, Hap_8, Hap_10, Hap_11, Hap_12, Hap_13, and Hap_14 were found in only one population, respectively. The shared haplotypes from COI marker represented 35.7% of the entire data set, while singleton haplotypes represented 64.3% (Table S2). Populations WGC, TWR, GNC, KQC, and AGT had the highest number of haplotypes from COI markers.

Compared to COI marker, the D-loop marker exhibited higher haplotype diversity. A total of 27 haplotypes were identified across the 11 populations. As shown in Table S2, Hap_8 was the most widespread haplotype, which was identified in 10 populations, followed by Hap_5 (nine populations), Hap_6 (nine populations), Hap_3 (eight populations), and Hap_4 (eight populations). The remaining haplotypes were found in only three or fewer populations. The shared haplotypes from D-loop marker represented 46.4% of the entire data set, while singleton haplotypes represented 53.6% (Table S2). TWR population had the highest number of haplotypes from D-loop markers. All haplotype sequences obtained from COI and D-loop markers are shown in Supplementary File “haplotype_seq.txt”.

As shown in Table 1, the average haplotype diversity (Hd) value for COI marker was 0.543, and for D-loop was 0.886. The haplotype diversity for COI marker among all populations ranged from 0.00 to 0.867, which were lower than that for D-loop marker (0.600–1.000). The average nucleotide diversity (π) was 0.0041 for COI markers, and 0.00436 for D-loop markers. The number of segregating sites in different populations ranged from 0 to 26 for COI markers, and ranged from 1 to 22 for D-loop markers. Among all populations, KSX had the highest genetic diversity, with the highest value of haplotype diversity and nucleotide diversity for both COI and D-loop markers, while ZLHX population had the lowest genetic diversity (Table 1).

### 3.2. Phylogenetic Analysis

**Figure 2:**
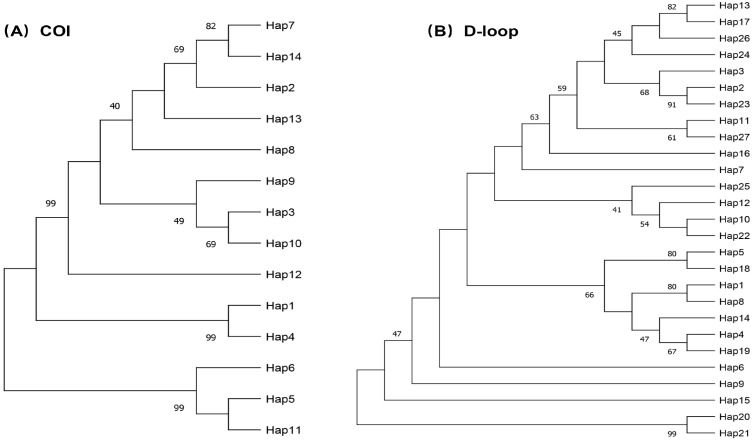
Phylogenetic trees of *Schizopygopsis pylzovi* constructed from COI and D-loop haplotypes. Numbers at the nodes represent bootstrap support values (only values > 40% are displayed).

The topologies built from the haplotype sequences of COI and D-loop region were dissimilar. Two phylogenetic trees showed that some haplotypes were weakly associated, with bootstrap support less than 40%, which was possibly due to low nucleotide acid variation (Figure 2). The haplotypes of COI gene revealed two clades, the upper branch contained most of haplotypes, the lower branch contained only three haplotypes. For D-loop region, the haplotypes were clustered into three branches.

**Figure 3:**
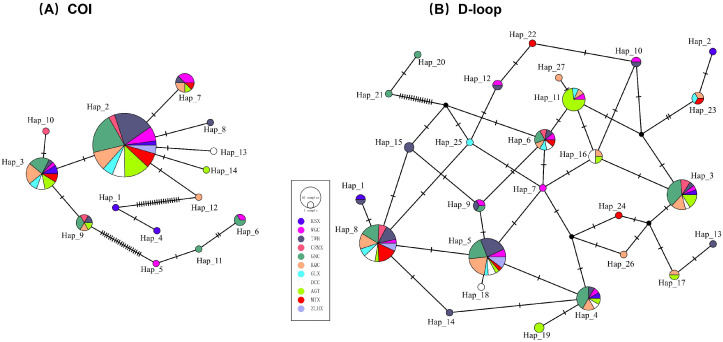
TCS networks of *Schizopygopsis pylzovi* based on COI and D-loop haplotypes.

The TCS networks were constructed from the haplotype sequences of the COI gene and D-loop region, respectively (Figure 3). The COI haplotype displayed a star-like topology, with Hap2 as the central core haplotype shared by the majority of populations, and several rare, closely related haplotypes radiating from it, suggesting a recent population expansion or bottleneck event followed by demographic recovery. The D-loop haplotype network was more complex, featuring multiple high-frequency haplotypes interconnected by several steps, suggesting a more ancient demographic history.

### 3.3. Genetic Structure and Population Differentiation

Molecular analysis of variance (AMOVA) was performed to evaluate the genetic structure and population differentiation based on COI and D-loop sequences. The results of molecular ANOVA carried out on COI and D-loop genes are shown in Table 2, which revealed different patterns of genetic partitioning between the two molecular markers. For the COI gene, genetic variation was mainly distributed within populations, accounting for 93.34% of the total variance, whereas variation among populations contributed only 6.66%. The fixation index (*F*_ST_) was 0.06658 with *p* < 0.05, indicating statistically significant, albeit low, genetic differentiation among populations. For the D-loop marker, it exhibited an even higher proportion of within-population genetic variance, reaching 97.43%, while among population variation accounted for merely 2.57%. The *F*_ST_ value was 0.02572 (*p* > 0.05). Further pairwise *F*_ST_ test was performed, and the results (Table S3) showed that, for the D-loop marker, the significant pairwise *F*_ST_ was observed between the population AGT and other five populations (*p* < 0.05), including TWR, GNC, DCC, MTX, and ZLHX; for the COI marker, the significant pairwise *F*_ST_ was observed between the population AGT and KSX, TWR and KSX, TWR and WGC (*p* < 0.05). Collectively, both COI and D-loop markers demonstrated that the majority of genetic variance resided within populations, accompanied by low *F*_ST_ values. The low mitochondrial differentiation is consistent with historical connectivity among populations.

### 3.4. Population Historic Dynamics

**Figure 4:**
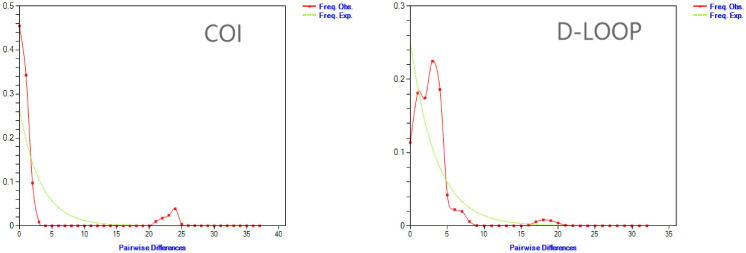
Mismatch distribution analysis of all *Schizopygopsis pylzovi* populations based on COI and D-loop sequences. The left panel is the mismatch distribution graph for COI, and the right panel is the mismatch distribution graph for D-loop. X-axis represents the number of pairwise differences. Y-axis represents the frequency of mismatches.

Neutrality tests (Tajima’s D and Fu’s Fs) and mismatch distribution analysis were performed based on COI and D-loop sequences to infer the historical population dynamics of the *S. pylzovi*. For COI gene marker, except two populations, KSX and GLX, showed positive Tajima’s D values, most of the examined populations exhibited negative Tajima’s D values, with a mean value of −0.418 (Table 1). The TWR population showed a significantly negative Tajima’s D (D = −1.679, *p* < 0.05) and AGT showed a strongly negative Tajima’s D (D = −1.196, *p* > 0.05), and this trend was further supported by Fu’s Fs test, with significantly negative values in both TWR (Fs = −3.273, *p* < 0.05) and AGT (Fs = −2.195, *p* < 0.05), indicative of population expansion. The ZLHX population showed zero values for Tajima’s D, Fu’s Fs, and also Hd, π, and S, suggesting a severe loss of genetic diversity consistent with a recent population bottleneck or strong artificial selection. Neutrality test results for the D-loop region differed from COI marker. As shown in Table 1, most of the examined populations exhibited negative Fu’s Fs values, with a mean value of −1.932. Fu’s Fs revealed highly significant negative values in WGC (Fs = −7.9, *p* < 0.05), TWR (Fs = −6.225, *p* < 0.05), KQC (Fs = −3.968, *p* < 0.05), and DCC (−2.707, *p* < 0.05) which, together with high haplotype diversity, implies that the D-loop region retains signals of more recent population expansion. The mismatch distribution analysis observed unimodal patterns in all populations for both COI (raggedness index = 0.2109, *p* = 0.5739; SSD = 0.0415, *p* = 0.1854) and D-loop markers (raggedness index = 0.1303, *p* = 0.5197; SSD = 0.0374, *p* = 0.3711) (Figure 4), suggesting a sudden demographic expansion, which was consistent with the Neutrality test results.

## 4. Discussion

Understanding the genetic diversity and population structure of endemic species is fundamental for their conservation and for deciphering the evolutionary history of plateau ecosystems. In this study, two mitochondrial molecular markers, COI and the D-loop region, were used to comprehensively assess the genetic characterization of 11 geographic populations of *S. pylzovi* from the upper Yellow River Basin. Our results revealed different patterns of genetic diversity between markers, low but significant population differentiation, and distinct signals of demographic history, highlighting the value of joint analysis using COI and D-loop markers in population genetics. These findings align with a previous study on schizothoracine fishes [20].

The present study revealed distinct genetic diversity profiles between the two mitochondrial markers. This difference is consistent with their well-documented evolutionary characteristics: the non-coding, fast-evolving nature of the D-loop accumulates mutations more rapidly than the protein-coding COI gene [21,22,23]. Relatively high haplotype diversity was observed in most D-loop populations, suggesting that this marker is well-suited for detecting recent population dynamics and fine-scale genetic structuring. Conversely, the COI gene, being more conserved, revealed lower haplotype diversity but captured a higher proportion of among population variation, indicating its utility for detecting deeper, albeit weaker, population differentiation. It is important to note that both COI and D-loop are mitochondrial DNA markers that follow strict maternal inheritance. This inheritance pattern means our interpretations of population structure only reflect the maternal lineage dynamics of *S. pylzovi*, which cannot infer the genetic differentiation of male individuals or paternal genetic contributions. To obtain a comprehensive view of the species’ overall population genetic structure, complementary nuclear molecular markers such as genome-wide SNPs should be adopted in follow-up studies to characterize both maternal and paternal genetic patterns.

Notably, a large number of singleton haplotypes were detected in both markers. This pattern, together with a star-like haplotype network with a dominant central haplotype surrounded by many low-frequency derived haplotypes, highly suggested this species had undergone rapid expansion or recovered from a bottleneck. New mutations have not yet had time to rise to high frequencies. The combination of high haplotype diversity and relatively low nucleotide diversity, especially in the D-loop, further reinforce the inference that the species has experienced recent demographic growth, likely following postglacial recolonization of the plateau. Moreover, the KSX population displayed the highest genetic diversity for both markers, accompanied by private haplotypes (e.g., Hap_1 and Hap_4 in COI) and isolated branching in the haplotype network (Figure 3). The KSX population is located at the border of Qinghai and Gansu provinces, where the Yellow River forms a large bend and flows through a mosaic of grasslands and gorges, featuring excellent water quality, abundant water resources, and unique wetland landscapes. We hypothesize that this habitat complexity likely contributed to the higher genetic diversity observed at this site, possibly in conjunction with localized geographic isolation [24]. In contrast, the ZLHX population showed very low genetic diversity for both markers, suggesting a severe genetic bottleneck or strong artificial selection [25]. This population is situated at the Yellow River source at an altitude over 4300 m, characterized by persistently low water temperatures and a short growing season. Such extreme high-altitude conditions may constrain population size and lead to genetic bottlenecks. The discrepancy between the COI (Hd = 0, π = 0, S = 0) and D-loop (Hd = 0.600, π = 0.00088, S = 1) markers in ZLHX arises from their different evolutionary rates: the conserved COI gene, which is under purifying selection, loses variation rapidly in a bottlenecked population, whereas the fast-evolving non-coding D-loop may still retain some rare genetic variants. It should be noted that the sample size of ZLHX are relatively small. The field access of this sampling site is extremely difficult. Exhaustive sampling was conducted in strict accordance with fishery management permits and wildlife protection regulations, yet no additional specimens could be collected. We must acknowledge that the small sample size for ZLHX could lead to an underestimation of haplotype diversity if rare haplotype remain undetected. Despite this limitation, the signal of genetic impoverishment in ZLHX appears robust: (1) For the COI marker, all four samples shared a single haplotype. If the population harbored even moderate polymorphism, the probability of observing complete monomorphism across four samples solely by chance would be low. (2) For the D-loop marker, it is also markedly lower than that of most other populations. Thus, although the possibility that a larger sample would reveal a few additional rare variants cannot be excluded, the overall results that ZLHX has low genetic diversity is credible, and the exact genetic diversity level of ZLHX needs to be further verified with expanded sampling in future studies.

Population structure analysis revealed that the vast majority of genetic variation resided within populations, with low but significant *F*_ST_ for COI and non-significant differentiation for D-loop in the AMOVA results. These findings indicated weak population structuring, consistent with the ecological characteristics of *S. phlzovi* as a widely distributed, highly mobile fish in the upper Yellow River [26]. The contrasting significance levels between the two markers are not contradictory but rather reflect their different evolutionary timescales. The conserved COI gene is more likely to retain signals of subtle, deeper population differentiation, whereas the fast-evolving D-loop may homogenize such faint structure more rapidly. This discrepancy further demonstrates the advantage of combining both markers, as the COI can detect weak historical differentiation that the D-loop alone might miss, while the D-loop captures fine-scale contemporary patterns. It is worth noting that this pattern dominated by intraspecific variation is consistent with the conclusions drawn by Chan et al. [27] in their analysis of the genetic structure of *Gymnocypris dobula*, and may reflect the synergistic shaping effect of large-scale geographical isolation and small-scale hydrological connectivity on the genetic structure of aquatic organisms. Despite overall weak structuring, the haplotype networks revealed geographic clustering: the COI network displayed a star-like topology centered on the widespread Hap_2, consistent with recent population expansion, while the D-loop network showed multiple interconnected high-frequency haplotypes with different populations exhibiting distinct haplotype assemblages. For instance, D-loop haplotypes Hap_20 and Hap_21 were largely restricted to the GNC population, which may be attributable to the influence of microgeographic barriers formed by the stepped uplift of the northeastern margin of the Tibetan Plateau [28], while Hap_3 spanned from KSX to MTX, supporting the hypothesis that localized hydrological features shape genetic structure [29].

Neutrality test results indicated that the *S. pylzovi* population exhibited different evolutionary dynamics between the COI and D-loop regions, with the COI gene being more suitable for detecting selection pressures in long-term evolutionary processes [30], while the D-loop region is more responsive to recent population dynamics. For the COI gene, most populations showed negative Tajima’s D values although non-significant, with TWR and AGT exhibiting negative values and further supported by significantly negative Fu’s Fs values. These results, together with the unimodal mismatch distributions, suggested historical population expansion or bottleneck recovery in the *S. pylzovi* population [31]. The mismatch distribution analysis showed that the overall population presented typical unimodal distribution curves, which is a classic signal of historical sudden population expansion. In contrast, the D-loop region yielded a mean Tajima’s D close to zero, but Fu’s Fs revealed highly significant negative values in WGC, TWR, KQC, and DCC. This discrepancy, with non-significant Tajima’s D but strongly negative Fu’s Fs, is a classic signature of recent rapid population expansion, as Fu’s Fs is more sensitive to recent demographic changes [16].

Taken together, these results suggested that *S. pylzovi* experienced historical or more recent localized population expansion. The high haplotype diversity coupled with expansion signals in most populations is consistent with the hypothesis that they originated from glacial refugia during Pleistocene climatic oscillations, followed by postglacial recolonization [32].

## 5. Conclusions

By integrating COI and D-loop markers, this study reveals high within-population genetic variation and weak differentiation in *S. pylzovi* across the upper Yellow River, which may suggest recent demographic expansions. The ZLHX population showed extremely low diversity, but given the small sample size, this pattern should be treated as a hypothesis for future testing. As our markers reflect only female-mediated genetic diversity, future work should incorporate nuclear genomic markers, expand sampling for under-represented populations, and integrate ecological data to clarify dispersal dynamics and guide conservation of this vulnerable plateau fish.

## Figures and Tables

**Table 1:** Genetic diversity and neutrality test of COI and D-loop gene of in 11 geographic populations of *Schizopygopsis pylzovi*. N: sample size; h: number of haplotypes; Hd: haplotype diversity; π: nucleotide diversity; S: number of segregating loci in different populations. Asterisks denote significant differences.

Populations	Genetic Diversity				Neutrality Test	
	N	Hd	π	S	Tajima’s D	Fu’s Fs
**COI**						
AGT	18	0.549	0.00104	4	−1.196	−2.195 *
CRMX	5	0.700	0.00207	3	−0.175	0.061
DCC	9	0.556	0.00091	2	−0.583	−0.532
GLX	7	0.476	0.00071	1	0.559	0.589
GNC	29	0.549	0.00741	25	−0.769	5.360
KSX	6	0.867	0.01788	23	1.243	2.995
KQC	18	0.693	0.00134	4	−0.673	−1.522
MTX	10	0.511	0.00082	2	−0.691	−0.594
TWR	23	0.324	0.00063	4	−1.679 *	−3.273 *
WGC	13	0.692	0.01060	26	−0.636	3.773
ZLHX	4	0	0	0	0	0
Total	142			40		
Mean		0.543	0.0041		−0.418	0.424
s.d.					0.805	2.623
**D-LOOP**						
AGT	18	0.791	0.00372	8	0.314	−1.675
CRMX	5	0.800	0.00411	5	1.124	1.220
DCC	11	0.909	0.00310	7	−0.477	−2.707 *
GLX	7	0.952	0.00448	7	0.345	−2.200 *
GNC	28	0.849	0.00647	22	−0.787	1.275
KSX	4	1.000	0.00808	10	0.083	−0.399
KQC	22	0.892	0.00395	9	0.304	−3.968 *
MTX	10	0.778	0.00424	8	0.093	−0.907
TWR	22	0.879	0.00338	10	−0.553	−6.225 *
WGC	11	0.982	0.00358	7	0.079	−7.900 *
ZLHX	5	0.600	0.00088	1	1.225	0.626
Total	143			27		
Mean		0.886	0.00440		0.140	−1.932
s.d.					0.609	2.914

**Table 2:** AMOVA analysis of COI and D-loop region sequences in *Schizopygopsis pylzovi*.

AMOVA Design and Results				
Source of Variation	d.f.	Sum of Squares	Variance Components	Percentage of Variation
**COI**				
Among populations	10	24.578	0.09288 Va	6.66
Within populations	131	170.57	1.30206 Vb	93.34
Total	141	195.148	1.39494	100
Fixation Index *F*_ST_	0.06658			
*p*-value	0.02835			
**D-LOOP**				
Among populations	10	19.518	0.03879 Va	2.57
Within populations	132	192.504	1.46949 Vb	97.43
Total	142	212.021	1.50829	100
Fixation Index *F*_ST_	0.02572			
*p*-value	0.07918			

## Data Availability

All data used in this study is deposited in GenBank, under accession number PZ484557-PZ484695 and PZ565047-PZ565189.

## Supplementary Materials

The following supporting information can be downloaded at: https://www.mdpi.com/article/10.3390/ani16131946/s1, Table S1: The detailed information for the 11 sampling sites; Table S2: Haplotype distribution of *Schizopygopsis pylzovi* across 11 geographic populations based on COI and D-loop markers; Table S3: Pairwise *F*_ST_ (below diagonal) and *p* values (above diagonal) for the COI and D-loop markers among 11 *Schizopygopsis pylzovi* populations; haplotype_seq.txt: All haplotype sequences obtained from COI and D-loop markers.

## References

[1] Li S., Gu H., Wang Y., Wang Z. (2023). Factors Limiting the Spread of Middle- and Low-Altitude Fishes to the Qinghai-Tibet Plateau. Front. Mar. Sci..

[2] Zhou C., Wang X., Hu Z., Chen Q., Du C., Liu Y., Song Z. (2023). Comparative Analyses Reveal Potential Genetic Mechanisms for High-Altitude Adaptation of *Schizopygopsis* Fishes Based on Chromosome-Level Genomes. J. Hered..

[3] Lai J., Liu Y., Chen Y., Li H., Du J., Li L. (2015). The Complete Mitochondrial Genome of *Schizopygopsis pylzovi* (Teleostei, Cyprinidae, *Schizopygopsis*). Mitochondrial DNA Part A.

[4] Ma Y.N., Du Y.Y., Zhang Y.P., Wang T. (2016). Population Genetic Structure and Its Implication in the Conservation of *Schizopygopsis pylzoviin* Yellow River as Inferred from Mitochondrial DNA Sequence Analysis. Genet. Mol. Res..

[5] Zhang W., Jiang S., Salumy K.R., Xuan Z., Xiong Y., Jin S., Gong Y., Wu Y., Qiao H., Fu H. (2022). Comparison of Genetic Diversity and Population Structure of Eight *Macrobrachium nipponense* Populations in China Based on D-loop Sequences. Aquac. Rep..

[6] Sato S., Kojima H., Ando J., Ando H., Wilmot R.L., Seeb L.W., Efremov V., LeClair L., Buchholz W., Jin D.-H. (2004). Genetic Population Structure of Chum Salmon in the Pacific Rim Inferred from Mitochondrial DNA Sequence Variation. Environ. Biol. Fish..

[7] Bronstein O., Kroh A., Haring E. (2018). Mind the Gap! The Mitochondrial Control Region and Its Power as a Phylogenetic Marker in Echinoids. BMC Evol. Biol..

[8] Zhao L., Chenoweth E.L., Liu Q. (2017). Population structure and genetic diversity of *Sinibrama macrops* from Ou River and Ling River based on mtDNA D-loop region analysis, China. Mitochondrial DNA Part A.

[9] Arai T., Taha H., Alidon N., Jumat J., Azmey S., Zan N.D., Jaafar T.N.A.M., Habib A. (2023). Mitochondrial Cytochrome c Oxidase Subunit I Gene Analysis of the Yellowfin Snapper, *Lutjanus xanthopinnisin* the Indo-Pacific Region and a Note on *Lutjanus* Population Structure. Heliyon.

[10] Lin M., Liang X.-F., Lu K., Zeng M., Gao J., Dou Y., Kuang Y., Zhang Q. (2025). Genetic Diversity in Three Sinipercine Fishes Based on Mitochondrial D-loop and COX1 Sequences. Fishes.

[11] Aminisarteshnizi M., Moyo N.A.G., Raphalo M.E. (2024). Genetic and Haplotype Diversity of Redbreast Tilapia (*Coptodon rendalli*) Based on Cytochrome Oxidase Subunit I and D-loop. J. King Saud Univ. Sci..

[12] Fang D., Luo H., He M., Mao C., Kuang Z., Qi H., Xu D., Tan L., Li Y. (2022). Genetic Diversity and Population Differentiation of Naked Carp (*Gymnocypris przewalskii*) Revealed by Cytochrome Oxidase Subunit I and D-loop. Front. Ecol. Evol..

[13] Ahmad Khan N., Naeem M. (2024). Efficiency evaluation of DNA isolation techniques in fins of Channa marulius using PCR amplification and NanoDrop. Momona Ethiop. J. Sci..

[14] Tamura K., Stecher G., Kumar S. (2021). MEGA11: Molecular Evolutionary Genetics Analysis Version 11. Mol. Biol. Evol..

[15] Rozas J., Ferrer-Mata A., Sánchez-DelBarrio J.C., Guirao-Rico S., Librado P., Ramos-Onsins S.E., Sánchez-Gracia A. (2017). DnaSP 6: DNA Sequence Polymorphism Analysis of Large Data Sets. Mol. Biol. Evol..

[16] Tran Lu Y A., Ruault S., Daguin-Thiébaut C., Le Port A.-S., Ballenghien M., Castel J., Gagnaire P.-A., Bierne N., Arnaud-Haond S., Poitrimol C. (2025). Comparative Population Genomics Unveils Congruent Secondary Suture Zone in Southwest Pacific Hydrothermal Vents. Mol. Biol. Evol..

[17] Leigh J.W., Bryant D. (2015). popart: Full-Featured Software for Haplotype Network Construction. Methods Ecol. Evol..

[18] Zhao F., Liu Y., Wang Z., Lu J., Cao L., Zeng C. (2023). Genetic Diversity and Connectivity of *Ocypode ceratophthalmus* in the East and South China Seas and Its Implications for Conservation. Biology.

[19] Guo S.S., Zhang G.R., Guo X.Z., Wei K.J., Yang R.B., Wei Q.W. (2014). Genetic Diversity and Population Structure of *Schizopygopsis younghusbandi Regan* in the Yarlung Tsangpo River Inferred from Mitochondrial DNA Sequence Analysis. Biochem. Syst. Ecol..

[20] Tong C., Fei T., Zhang C., Zhao K. (2017). Comprehensive Transcriptomic Analysis of the Tibetan Schizothoracinae Fish *Gymnocypris przewalskii* Reveals How It Adapts to a High Altitude Aquatic Life. BMC Evol. Biol..

[21] Peng M., Zhu W., Yang C., Yao J., Chen H., Jiang W., He Z., Li Q., Liu Q., Zhao Y. (2020). Genetic Diversity of Mitochondrial D-LOOP Sequences in the Spotted Scat (*Scatophagus argus*) from Different Geographical Populations along the Northern Coast of the South China Sea. J. Appl. Ichthyol..

[22] Zhang J.J., Duan J.R., Zhou Y.F., Peng J.Y., Fang D.A. (2017). Genetic diversity of mitochondrial control region (D-Loop) polymorphisms in *Coilia ectenes taihuensis* inhabiting Taihu Lake, China. Genet. Mol. Res..

[23] Feng X., Wang X., Zhu R., Jia Y., Sui X., Zhuo Y., Li J., Chen Y. (2025). Genetic Diversity and Population Genetic Structure of Endemic Schizothoracinae Fishes in the Upper Yellow River and Its Adjacent Waters. Ecol. Evol..

[24] Zhou Y., Lei Y., Lu Y., Song Z. (2018). Population Genetics of a Chinese Endemic, *Gymnocypris potanini* Herzenstein, Threatened by Population Isolation: Conflicting Patterns Between Microsatellites and Mitochondrial DNA. Hydrobiologia.

[25] He J., He Z., Yang D., Ma Z., Chen H., Zhang Q., Deng F., Ye L., Pu Y., Zhang M. (2022). Genetic Variation in *Schizothorax kozlovi* Nikolsky in the Upper Reaches of the Chinese Yangtze River Based on Genotyping for Simplified Genome Sequencing. Animals.

[26] Zhou C., Zhou Y., Xu L., Liu F., Lei L., Gao H., Li J., Fu S., Duan Y., Tan Y. (2024). Chromosome-Level Genome Assembly and Population Genomic Analysis Provide Insights into the Genetic Diversity and Adaption of *Schizopygopsis younghusbandi* on the Tibetan Plateau. Integr. Zool..

[27] Chan J., Li W., Hu X., Liu Y., Xu Q. (2016). Genetic Diversity and Population Structure Analysis of Qinghai-Tibetan Plateau Schizothoracine Fish (*Gymnocypris dobula*) Based on mtDNA D-loop Sequences. Biochem. Syst. Ecol..

[28] Guo J., Xie X., Sun H., Wang A., Shi Z., Li X. (2025). Tibetan Plateau Uplift Intensified Aridity in Inland Asia: The Role of the Dust-Ice Cloud Interaction Feedback Mechanism. Palaeogeogr. Palaeoclimatol. Palaeoecol..

[29] Porto-Hannes I., Burlakova L.E., Lasker H.R. (2022). Genetic Isolation and Homogenization: Potential Effects of Landscape Features on the Population Genetic Structure of Freshwater Mussels. J. Great Lakes Res..

[30] Xu L., Li H., Wang L., Du F. (2018). Genetic structure and haplotype pattern of marine planktonic ostracod (*Porroecia spinirostris*) from South China Sea based on mitochondrial COI gene. Ocean Sci. J..

[31] Liu B., Zhang K., Zhu K., Shafi M., Gong L., Jiang L., Liu L., Muhammad F., Lü Z. (2020). Population Genetics of *Konosirus punctatus* in Chinese Coastal Waters Inferred from Two mtDNA Genes (COI and Cytb). Front. Mar. Sci..

[32] Parvizi E., Keikhosravi A., Naderloo R., Solhjouy-Fard S., Sheibak F., Schubart C.D. (2019). Phylogeography of *Potamon ibericum* (Brachyura: Potamidae) Identifies Quaternary Glacial Refugia Within the Caucasus Biodiversity Hot Spot. Ecol. Evol..

