# Supplementary material for: Genetic and Haplotype Diversity of Schizopygopsis pylzovi in the Yellow River on the Northeastern Qinghai–Tibet Plateau
Source: Animals (Basel). 2026 Jun 23;16(13):1946. doi: 10.3390/ani16131946 (PMC13360073; doi:10.3390/ani16131946)
Supplement: Supplementary file 1 [file animals-16-01946-s001.zip › Supplementary Tables.pdf]

Table S1. The detailed information for the 11 sampling sites.

Sampling Sites	Longitude (°E)	Latitude (°N)	Province	Township/village	Sampling time
KSX	101.662	34.118	Qinghai	Kesheng Township	2025.06
WGC	102.323	33.649	Gansu	Wenge Village	2025.06
TWR	101.896	34.005	Gansu	Taiwu Ruo Village	2025.06
CRMX	102.082	33.331	Gansu	Cairima Township	2025.06
GNC	101.736	33.711	Gansu	Gongnai Village	2025.06
KQC	99.156	33.882	Qinghai	Kequ Village	2025.06
GLX	100.321	33.759	Qinghai	Ganglong Township	2025.06
DCC	99.722	33.815	Qinghai	Dangcheng Village	2025.06
AGT	101.141	34.482	Qinghai	Agatang Village	2025.06
MTX	101.03	33.796	Qinghai	Mentang Township	2025.06
ZLHX	97.924	35.083	Qinghai	Zhalinghu Township	2025.06

Table S2. Haplotype distribution of *Schizopygopsis pylzovi* across 11 geographic populations based on COI and D-loop markers.

	Haplotype-based genetic statistics										
	**KSX**	**WGC**	**TWR**	**CRMX**	**GNC**	**KQC**	**GLX**	**DCC**	**AGT**	**MTX**	**ZLHX**
**COI**											
Hap_1	1										
Hap_2	2	7	19	3	19	9	5	6	12	7	4
Hap_3	2	1	1		5	5	2	2	3	2	
Hap_4	1										
Hap_5		1									
Hap_6		1			2						
Hap_7		3	1			2			1	1	
Hap_8			1								
Hap_9			1	1	2	1			1		
Hap_10				1							
Hap_11					1						
Hap_12						1					
Hap_13								1			
Hap_14									1		
Total	6	13	23	5	29	18	7	9	18	10	4
**D-LOOP**											
Hap_1	1		1								
Hap_2	1										
Hap_3	1	1	1	2	7	3		1	3		
Hap_4	1	1	1		5	2		1	1		
Hap_5		2	7		6	6	1	2	1	1	3
Hap_6		1	1	1	2	1	1	1		1	
Hap_7		1									
Hap_8		1	4	2	5	4	2	3	1	5	2
Hap_9		1	1		1						
Hap_10		1	1								
Hap_11		1				1	1		8		
Hap_12		1	1								
Hap_13			1								
Hap_14			1								
Hap_15			2								
Hap_16						1		2	1		
Hap_17						1			1		
Hap_18								1			
Hap_19									2		
Hap_20					1						
Hap_21					1						
Hap_22										1	

Hap_23						1	1			1	
Hap_24										1	
Hap_25							1				
Hap_26						1					
Hap_27						1					
Total	4	11	22	5	28	22	7	11	18	10	5

Table S3. Pairwise F_{ST} (below diagonal) and *p* values (above diagonal) for the COI and D-loop markers among 11 *Schizopygopsis pylzovi* populations.

	Populations	KSX	WGC	TWR	CRMX	GNC	KQC	GLX	DCC	AGT	MTX	ZLHX
**DLOOP**	**KSX**		0.5045	0.1171	0.9730	0.5225	0.6036	0.6757	0.3063	0.3423	0.5045	0.0901
	**WGC**	-0.0302		0.3063	0.7117	0.6036	0.6487	0.9369	0.8198	0.0901	0.5315	0.1081
	**TWR**	0.0736	0.0154		0.3784	0.2883	0.0811	0.3153	0.5496	0.0000	0.6937	0.7478
	**CRMX**	-0.0152	-0.0675	0.0188		0.7658	0.8108	0.8919	0.7027	0.3333	0.7207	0.1712
	**GNC**	-0.0478	-0.0168	0.0106	-0.0699		0.6396	0.4775	0.8919	0.0180	0.3604	0.3514
	**KQC**	-0.0558	-0.0228	0.0368	-0.0686	-0.0086		0.3784	0.7928	0.0631	0.1892	0.0631
	**GLX**	-0.0861	-0.0779	0.0083	-0.1267	-0.0201	-0.0029		0.5405	0.1261	0.8378	0.1892
	**DCC**	-0.0020	-0.0572	-0.0140	-0.0647	-0.0379	-0.0424	-0.0214		0.0451	0.4775	0.3423
	**AGT**	0.0205	0.0667	0.2280*	0.0238	0.0908*	0.0601	0.0696	0.1076*		0.0090	0.0090
	**MTX**	-0.0388	-0.0092	-0.0283	-0.0789	-0.0028	0.0246	-0.0812	-0.0009	0.1859*		0.2072
	**ZLHX**	0.1667	0.1219	-0.0541	0.1827	-0.0046	0.0987	0.1264	0.0447	0.3557*	0.0234	
**COI**	**KSX**		0.1351	0.0000	0.4234	0.0631	0.0631	0.2883	0.1351	0.0451	0.1081	0.3153
	**WGC**	0.0965		0.0451	0.4324	0.5315	0.1261	0.3694	0.3694	0.0721	0.4324	0.6667
	**TWR**	0.4550*	0.1192*		0.1622	0.0811	0.1261	0.3874	0.5405	0.4595	0.7027	0.9910
	**CRMX**	0.1308	-0.0318	0.1428		0.8469	0.6036	0.7297	0.2883	0.4505	0.2973	0.3964
	**GNC**	0.1826	-0.0357	0.0604	-0.0604		0.2342	0.4685	0.5405	0.1622	0.2072	0.3964
	**KQC**	0.3631	0.0744	0.0580	-0.0491	0.0262		0.8288	0.5315	0.8829	0.8469	0.3874
	**GLX**	0.2042	0.0060	0.0100	-0.0654	-0.0266	-0.0787		0.9910	0.9910	0.9910	0.4775
	**DCC**	0.2519	0.0293	-0.0010	-0.0139	-0.0071	-0.0308	-0.1079		0.9099	0.8919	0.6937
	**AGT**	0.3807*	0.0846	-0.0064	-0.0094	0.0326	-0.0294	-0.0852	-0.0539		0.9910	0.7568
	**MTX**	0.2746	0.0336	-0.0301	0.0094	0.0071	-0.0493	-0.0969	-0.0710	0.0689		0.6036
	**ZLHX**	0.1067	-0.0581	-0.1255	0.0698	-0.0619	0.0160	0.0572	-0.0435	0.0572	0.0582	
